# BAFF Blockade Attenuates DSS-Induced Chronic Colitis *via* Inhibiting NLRP3 Inflammasome and NF-κB Activation

**DOI:** 10.3389/fimmu.2022.783254

**Published:** 2022-03-07

**Authors:** Ying Zhang, Meihui Tao, Chaoyue Chen, Xi Zhao, Qinyu Feng, Guang Chen, Yu Fu

**Affiliations:** ^1^ Department of Gastroenterology, Union Hospital, Tongji Medical College, Huazhong University of Science and Technology, Wuhan, China; ^2^ Department of Integrated Traditional Chinese and Western Medicine, Tongji Hospital, Tongji Medical College, Huazhong University of Science and Technology, Wuhan, China

**Keywords:** B cell activating factor, inflammatory bowel disease, inflammation, macrophages, inflammasome

## Abstract

**Background:**

BAFF production is increased in IBD patients. However, the specific role of BAFF in IBD is still uncovered. This study aimed to investigate the expression and function of BAFF in experimental colitis and the potential mechanisms.

**Methods:**

BAFF levels in the serum and colon tissues were measured by ELISA in DSS-induced colitis mice. Mouse-derived BAFF antibody was administered in DSS mice. The changes of body weight, disease activity index (DAI) scores, colon length, spleen weight, histopathological damage, inflammatory indicators, NF-κB signaling, and NLRP3 inflammasome were assayed in DSS mice and control. LPS-primed RAW264.7 cells and bone marrow derived macrophages (BMDMs) were treated with BAFF blockage and recombinant mouse BAFF. Inflammatory associated cytokines, NLRP3 inflammasomes and NF-κB signaling were detected among groups.

**Results:**

BAFF production was elevated systemically and locally in colitis mice. BAFF blockade improved the body weight loss, DAI scores, colon length, spleen weight, and histopathological damage in colitis mice. Immunoflurescence analysis revealed that elevated macrophages in mucosal lamina propria were the primary source of BAFF in the colon. NLRP3 inflammasome and NF-κB signaling pathway activation were dramatically inhibited in DSS mice treated with BAFF blockage. In LPS-primed RAW264.7 cells/BMDMs, BAFF blockade decreased the activation of NLRP3 inflammasome (NLPR3, ASC, cleaved IL-1β, cleaved caspase 1) *via* inhibiting NF-κB signaling pathway. Moreover, LPS synergizes with BAFF to promote inflammatory factor secretion and expression of NF-κB signaling pathway in RAW264.7 cells.

**Conclusions:**

These results suggested that BAFF blockade protected against colitis partially by relieving inflammation, inhibiting intestinal NLRP3 inflammasome and NF-κB signaling pathway from macrophages. BAFF plays an important role in inflammation regulation in IBD, thus providing a novel idea for further research on colitis and experimental evidences for novel potential therapeutic target in IBD.

## Introduction

Inflammatory bowel disease (IBD) is a chronic disorder characterized by life-long treatment and incurable idiopathic intestinal inflammation, affecting the health and well-being of patients significantly (1). The etiology of IBD has not yet fully been elucidated and is associated with a variety of factors, namely, environmental, genetic, gut flora and immune factors, with immune dysregulation playing the key essential role. A variety of cytokines, namely, IL-1β, TNF-α, and IL-6 mediate intestinal inflammation in IBD, while relevant biologic agents have been developed such as anti-TNF drugs infliximab, IL-12/IL-23p40 antibody ustekinumab, integrin α4/β7 heterodimer monoclonal antibody vedolizumab, but their current widespread use is limited by several complex reasons, thus the inflammatory process in IBD needs further elucidation (2).

B cell-activating factor (BAFF) is a member of the tumor necrosis factor (TNF) super family, mainly expressed in cytoplasm from myeloid cells, but also found in other cells such as epithelial cells (respiratory tract, skin, etc.), mainly binding to three receptors: BAFF-receptor (BAFF-R), B cell maturation antigen (BCMA), transmembrane activator and calcium-modulating, and cyclophilin ligand interactor (TACI) and activating downstream signaling pathways. BAFF has gained significant attention in maintaining B cell survival, T cell, and B cell differentiation. Increasing studies hint BAFF may have an important regulatory role in inflammatory diseases, namely, systemic lupus erythematosus (SLE) (3), primary Sjögrens syndrome (pSS) (4), atherosclerosis (5), chronic obstructive pulmonary disease (COPD) (6), and IBD (7). Our previous study found that BAFF, a new biomarker for IBD, was strongly associated with IBD-related intestinal inflammation and that serum BAFF levels in patients with ulcerative colitis were strongly correlated with Mayo score and TNF-α presented all strong positive correlations (8). Previously, we detected increased expression of BAFF in serum and colon in IBD patients, with sensitivity, specificity and AUC of 88%, 100% and 0.936 in UC patients compared to irritable bowel syndrome (IBS) patients or healthy controls, respectively (8). Fodor in his latest study examined BAFF and fecal calprotectin in the stools of children with CD, children with UC, children with irritable bowel syndrome (IBS) and healthy children, discovered a significant increase in fecal BAFF concentration in group with IBD and a remarkable correlation between BAFF and fecal calprotectin (9). These studies suggest that BAFF may be a new non-invasive biomarker for screening patients with IBD and potentially being an effective biological target. Nonetheless, specific roles of BAFF in the immunopathogenesis of inflammatory bowel disease remain largely unknown.

Nucleotide-binding oligomerization domain-like receptors (NLRs/inflammasomes) are a subclass of the pattern recognition receptors (PRRs) family, and include NLRP3, NLRP6, NLRC4, AIM2, and NLRP6. Inflammasomes are expressed in monocytes, dendritic cells, eosinophils and other immune and non-immune cells such as fibroblasts and cardiomyocytes. Inflammasomes play an important role in maintaining intrinsic immunity and inflammatory homeostasis in response to pathogens and other inflammatory cytokines, and inducing pyroptosis (a form of programmed cell death). Inflammasomes are associated with the development of a variety of inflammatory and autoimmune diseases, namely, Alzheimer’s disease, hypertension, diabetes mellitus, atherosclerosis, asthma, IBD and so on. Inflammasomes are cytoplasmic sensors that recognize pathogenic infection, tissue damage or metabolic imbalance, which on activation lead to the maturation and release within a variety of pro-inflammatory factors, namely, interleukin-1β (IL-1β) and interleukin-18 (IL-18) (10–12). Two signals are generally needed for the complete activation of NLRP3 inflammasome. The first signal is induced by pathogen-associated molecular patterns (PAMP) through TLRs, by activating NF-κB following pro-IL-1β and NLRP3 synthesis. Second signal is the damage-associated molecular pattern (DAMP), NLRP3 recruit apoptosis-associated speck-like protein (ASC) and caspase-1, contributing to the self-activation with caspase-1 and the cleavage with pro-IL-1β into bioactive mature IL-1β. Lipopolysaccharide (LPS) at high doses (e.g., 1 ug/ml) is proven to provide precursor and activation signals to both human monocytes and macrophages, causing full-fledged IL-1β secretion (13). Studies currently show that blockade of NLRP3 reduces IL-1β maturation and inflammation (14). Recent decades have reported abundant evidence confirming the nucleotide-binding domain-like receptors family pyrin domain containing 3 (NLRP3) inflammasome plays an important role in IBD pathogenesis (15). Others showed inhibiting IL-1β or caspase-1 effectively improved intestinal inflammation in colitis model (16). NLRP3 deficient mice are susceptible to DSS-induced colitis induction and *NLRP3^−/−^* mice are prone to develop more severe colitis compared to controls responding to *C. rodentium* infection. *Pycard^−/−^* (ASC deficient) and *Caspase1^−/−^* mice are more susceptible to mortality from severe DSS-induced colitis and have a significantly higher risk of inflammation-associated colorectal cancer compared to wild-type mice (17–19). Furthermore, DSS has been shown to directly activate NLRP3 inflammasome and promote maturation of IL-1β, inducing intestinal inflammation. BAFF-BAFFR binding has been found to promote the assembly of NLRP3 inflammasome (NLRP3 and cleaved-IL-1β protein expression is upregulated) in B cells with dose-time dependent effects (20). Therefore, we explored whether BAFF regulates inflammasomes in IBD and affects intestinal inflammation and attempted to investigate the expression and function of BAFF in DSS-induced colitis models and identify the underlying mechanisms *in vivo* and *in vitro*.

## Materials & Methods

### Animals

Male C57BL/6J mice (6–8 weeks, 20–22 g) were purchased from the Beijing Vital River Laboratory Animal Technologies Co. Ltd. All mice were fed under specific pathogen free conditions in the animal facility of Tongji Medical College with free access to sterilized pure water and food. The mice were housed for at least one week at room temperature (25°C) with 12 h light/dark cycles for climatization before commencement of experiments. Animal experiments were approved by the Animal Management and Use Committee of Huazhong University of Science and Technology (S2526).

### Experimental Design

Mice were randomly divided into three groups: (1) Control group was treated by intraperitoneal injection of 200 ul sterile PBS (n = 6), (2) DSS-induced colitis group treated with mouse immunoglobulin G1 (IgG1) isotype control antibody (Adipogen Life Sciences) as a control (n = 9), and (3) DSS-induced colitis treated with mouse BAFF monoclonal antibody (Sandy-2, Adipogen Life Sciences) (n = 8). To induce chronic colitis, mice were challenged with 2.5% (w/v) DSS (MW 36,000–5,000 Da; MP Biomedicals, USA).Approximately 2.5% DSS drinking water in 4 cycles with drinking periods from 1 to 5 days, 8 to 12 days, 15 to 19 days and 22 to 26 days, and distilled water was drunk during the remaining period, mouse BAFF monoclonal antibody (2 mg/kg) or mouse immunoglobulin G1 (IgG1) isotype control antibody (2 mg/kg) was injected on the 1st and 15th days during molding to observe colitis.

During the course of the experiment, the body weight, stool consistency and bleeding were measured every day to assess the disease activity index (DAI). Briefly, DAI scores were defined as follows, for weight loss, 0: no loss; 1: 1–5% loss; 2: 5–10% loss; 3: 10–20% loss and 4: >20% weight loss; for stool consistency, 0: normal; 2: loose stool; 4: diarrhea; and for stool bleeding, 0: no blood; 2: presence and 4: gross blood (21).

LPS-induced murine macrophages cell line (RAW264.7 cells) or bone marrow-derived macrophages (BMDMs) with or without anti-BAFF monoclonal antibody, mouse immunoglobulin G1 (IgG1) isotype control antibody or recombinant mouse BAFF to discover whether BAFF regulate NF-κB signaling pathway and NLRP3 inflammasome.

### Quantitative Real-Time PCR

According to the manufacturer’s instructions, total RNA was extracted using the RNAiso plus (Takara, Japan). Its quantity and quality were measured by a NanoVue spectrophotometer (GE Healthcare, NJ), with 260/280 ratio of 1.8–2.0. Reverse transcribed into cDNA using the PrimeScript™ RT reagent Kit (Takara, Japan) following the manufacturer’s instructions. Quantitative PCR analyses were performed on a LightCycler^®^ 480 System (Roche) using the SYBR Green PCR Master Mix (Takara, Japan). The relative amount of RNA for each gene was normalized against β-actin (as the housekeeping gene) and analyzed using the 2^−ΔΔCt^ method. Amplification procedures were: pre-denaturation at 95°C for 10 min, and 40 cycles of denaturation at 95°C for 30 s, annealing at 60°C for 1 min, and extension at 72°C for 30 s. Primers designed to detect the gene expression of inflammatory cytokines, primers used in this experiment were: *Mouse NLRP3:5’-AGGCTGCTATCTGGAGGAACT-3’ forward; and 5’-CCTTTCTCGGGCGGGTAATC-3’ reverse. Mouse NLRP1:5’-CCATAGAGGAGCAGGCAGGTC-3’ forward; and 5’-TTGGGTCCACATCCTCTTGAC-3’ reverse. Mouse NLRP6:5’-TGAGACCAGTTTAGCCCAGA-3’ forward; and 5’-CTGGCACTGGCTCATAGAAC-3’ reverse. Mouse NLRC4:5’-CAGGTCACAGAAGAAGACCTGA-3’ forward; and 5’-ACTTCCCTTTGCCAGACTCG-3’ reverse. Mouse AIM2:5’-GCCGCCATGCTTCCTTAACT-3’ forward; and 5’-CTGTCTTGTTCCCACTGCCT-3’ reverse. Mouse IL-1β:5’ -CAGGCAGGCAGTATCACTCATTG-3’ forward; and 5’-CGTCACACACCAGCAGGTTATC-3’ reverse. Mouse IL-6:5’-GAGAAAAGAGTTGTGCAATG-3’ forward; and 5’-ATTTTCAATAGGCAAATTTC-3’ reverse. Mouse TNF-a: 5’-AGCCGATGGGTTGTACCT-3’ forward; and 5’-TGAGTTGGTCCCCCTTCT-3’ reverse. Mouse IL-18:5’-GCCTGTGTTCGAGGATATGACT-3’, forward; 5’-CCTTCACAGAGAGGGTCACAG-3’, reverse. Mouse β-actin:5’-AGTGTGACGTTGACATCCGTA-3’ forward;and 5’-GCCAGAGCAGTAATCTCCTTCT-3’ reverse*.

### Histopathological Examinations

Small segments of distal colon specimens were obtained and fixed with 4% paraformaldehyde (Servicebio, Wuhan) for 24 h at room temperature. Collected tissue samples were then embedded in paraffin, serially sectioned at 5 µm thick sections and stained section with hematoxylin and eosin (H&E) for morphological analysis. The histological analysis was performed as previously described (22). In brief, the sections were graded as to inflammation severity, inflammation extent and crypt damage scores were calculated by multiplying the score for these grades by their percentage involvement, giving a minimum score of 0 and a maximum score of 40: cellular infiltration (0–5), deterioration of crypt architecture (crypt damage, 0–4), extent of mucosal ulceration (0–3), and absence or presence of submucosal edema (0 and 1). Our histopathological scores were evaluated by two doctors of clinical pathology who were blinded to the group.

### Immunofluorescence Staining

To localize the expression of BAFF, paraffin section of colonic tissues (5 μm) were dewaxed, rehydration and conducted antigen heat retrieval in citrate buffer followed by blocking with 10% donkey serum for 30 min at room temperature. Next, sections were incubated with overnight with anti-BAFF antibody (1:100 dilution, ABclonal Technology) and F4/80 antibody (7.5 ug/ml, Abcam) overnight at 4°C. After washing with PBS for 3 times, slides were stained with F4/80 corresponding fluorescent secondary antibody anti-Rat (1:100 dilution, ABclonal Technology) and BAFF corresponding fluorescent secondary antibody anti-Rabbit (1:500 dilution, Abcam) for 1 h at room temperature. Sections were washed three times and nuclei were stained with 4′,6-diamidino-2- phenylindole (DAPI) (AntGene Biotechnology) for 10 min at room temperature. Images were captured using confocal laser scanning microscope (Olympus).

### Enzyme-Linked Immunosorbent Assay (ELISA)

BAFF concentrations in sera, tissue homogenates or cell supernatants were detected using the mouse BAFF quantikine ELISA kit (R&D systems) following the manufacturer’s instructions. Concentrations of interleukin 18 (IL-18) was also measured using ELISA kits (Bioswamp). ELISA for the determination of IL-18 in cell supernatant or mouse serum. The quantitative range of mouse BAFF quantikine ELISA kit was between 8 and 781 pg/ml. The quantitative range of mouse IL-18 quantikine ELISA kit was between 10 and 800 pg/ml. Samples from tissues, sera or cell supernatants were diluted 2–4 times for measuring.

### Western Blot

Total protein was extracted from intestinal tissues or cell lysates using radioimmunoprecipitation assay (RIPA) buffer (Beyotime, Biotechnology), which contained a protease and phosphatase inhibitor Cocktail (MedChemExpress). Proteins were quantified using a bicinchoninic acid (BCA) protein assay kit (Vazyme). Protein lysates were separated by 10 or 15% sodium dodecyl sulfate polyacrylamide gel electrophoresis (SDS-PAGE) (Shanghai EpiZyme Biotechnology Co.) then transferred onto polyvinylidene diflfluoride membranes (Millipore Corp., MA, USA). Membranes were blocked with blocking buffer composed of 5× rapid closure solution (Shanghai EpiZyme Biotechnology Co.) in Tris-buffered saline with 0.1% Tween-20 for 15 min at room temperature. Membranes were then incubated with primary antibodies at 4 °C for overnight and horse radish peroxidase (HRP) conjugated secondary antibodies (Cell Signaling Technology, USA). Proteins were visualized using an electrochemical luminescence (ECL) kit (Thermo Fisher Scientific) and chemiluminescence imaging system (Bio-Rad). The following primary antibodies were used: anti-NLRP3 (Cell Signaling Technology, USA), anti-ASC (Cell Signaling Technology, USA), anti-cleaved-IL-1β (Cell Signaling Technology, USA), anti-cleaved-caspase-1 (Cell Signaling Technology, USA), anti-*p*-IKKα/β (Cell Signaling Technology, USA), anti-IKKα/β (Cell Signaling Technology, USA), anti-*p*-p65 (Cell Signaling Technology, USA), anti-p65 (Cell Signaling Technology, USA), anti-*p*-IKBα (Cell Signaling Technology, USA), anti-IKBα (Cell Signaling Technology, USA) and glyceraldehyde-phosphate dehydrogenase (GAPDH) (Antgene Biotechnology).

### Cell Cultures

Mouse bone marrow-derived macrophages (BMDMs) were acquired as follows, femurs and tibiae of male C57BL/6 mice were rinsed with PBS, which contained 3% fetal bovine serum (FBS, Gibco, USA). Then supplemented with RPMI 1640 supplemented with 10% FBS, 100 U/ml penicillin–streptomycin (Gibco, USA) and 30 ng/ml M-CSF (Peprotech, USA) medium (Gibco, USA) to culture the isolated macrophages for 7 days. Change the fluid in half volume every other day. Full fluid change on day 7 to remove non-adherent cells and adherent cells were collected for the subsequent further experiments.

Mouse macrophage cell lines (RAW 264.7) were incubated in Dulbecco’s Modified Eagle’s Medium (Gibco, USA) supplemented with 10% FBS and 100 U/ml penicillin–streptomycin (Gibco, USA). After 80–90% confluency was reached, cells were removed from the flask substrate with a cell spatula. Cells were cultured in a cytoculture incubator (37°C and 5% CO2).

Subsequently, cells were intervened with 1 ug/ml LPS, 500 ng/ml or 1 ug/ml mouse BAFF monoclonal antibody (Sandy-2, Adipogen Life Sciences). LPS treatment was performed with anti-BAFF incubation simultaneously. Mouse immunoglobulin G1 (IgG1) isotype control antibody or 250 ng/ml of recombinant mouse BAFF (R & D Systems) was used in 6 or 12 well culture plates with or without LPS pretreatment for 6 h simultaneously with LPS, pulsed with ATP (2mM, Sigma Aldrich, USA) for 30 min. Cells or culture medium extract (23) were collected for further analysis.

### Statistical Analysis

GraphPad Prism 8.0 was used for statistical analysis. Data were presented as mean ± SEM. Differences among groups were compared using One-way-analysis of variance (ANOVA) or Mann–Whitney U test. One-way ANOVA was used for normally distributed data and Mann–Whitney U test was used for non-normally distributed data. P-value <0.05 was considered statistically significant.

## Results

### BAFF Expression is Systemically and Locally Upregulated in DSS-Induced Chronic Mice

**Figure 1:**
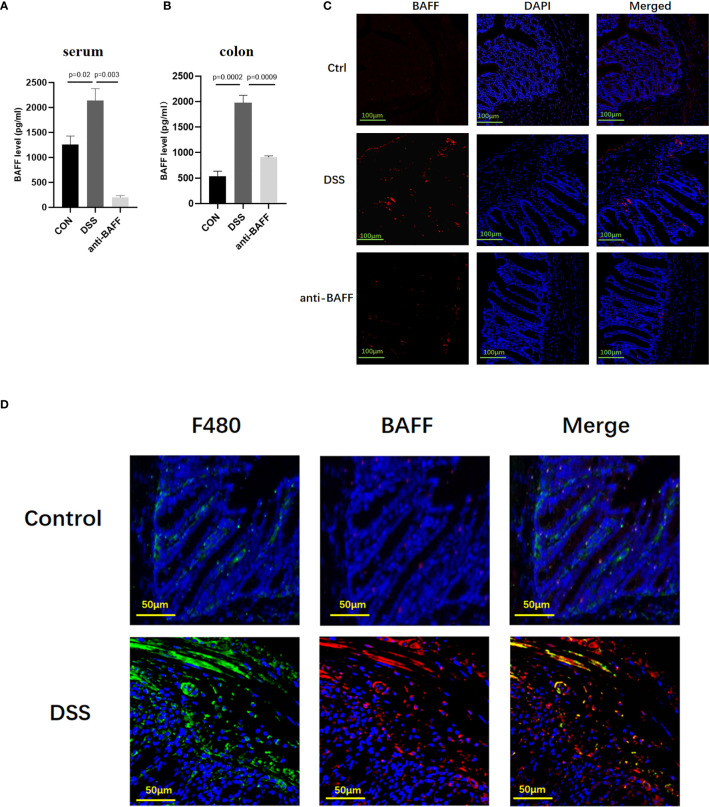
Systemic and local BAFF expression in DSS-induced colitis mice and distribution in intestinal mucosa. To induce chronic colitis, mice were challenged with 2.5% DSS; 5 days of 2.5% DSS, 2 days of sterile water, four cycles. Mice were injected intraperitoneally with BAFF monoclonal antibody (2 mg/kg) on days 1 and 15, while mice in the model group were injected intraperitoneally with IgG1 negative isotype control antibody (2 mg/kg) on days 1 and 15 throughout the modeling period. Blood samples and colon were collected at the indicated time points. **(A, B)** Samples were assayed for BAFF concentrations by ELISA. **(C)** Immunofluorescence of BAFF and DAPI staining in the colon tissues (n = 6–9 per group). **(D)** Dual-colored immunofluorescence staining was used to determine the expression of F4/80 (specific for monocytes/macrophages) (green fluorescence) and the expression of BAFF (red fluorescence). Ctrl, control.

Previous studies have reported that BAFF is elevated in the serum and colonic biopsy specimens of IBD patients (8). Here we analyzed BAFF expression in the established experimental mouse DSS-induced chronic colitis model using ELISA. In the DSS-induced mice colitis model, BAFF levels are significantly upregulated in the serum and colon (**Figures 1A, B**). Immunofluorescence showed that BAFF^+^ cells in the colon of DSS mice remarkably increased compared with control mice with a distribution pattern in lamina propria infiltrating mononuclear cells, especially in macrophages (**Figures 1C, D**). In anti-BAFF antibody group, BAFF concentrations in the serum and colon were extremely low, indicating that BAFF was successfully neutralized. Therefore, these findings indicated that the dysregulated expression of BAFF in murine intestine may be associated with DSS-induced colitis.

### BAFF Blockade Improves DSS-Induced Chronic Colitis

**Figure 2:**
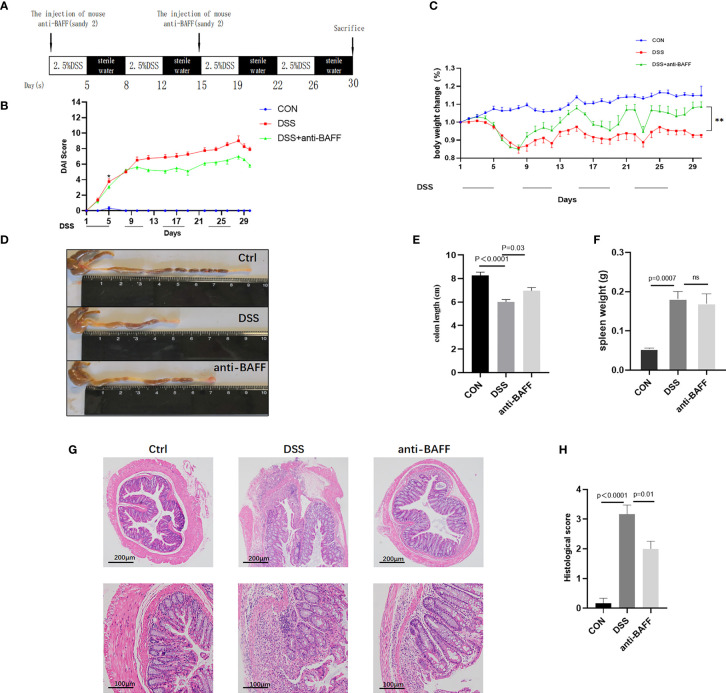
BAFF blockade ameliorates DSS-induced experimental chronic colitis in mice. **(A)** Detailed process and dosage regimen of DSS and anti-BAFF antibody. **(B)** Disease activity index was evaluated as the combined score of weight loss, fecal consistency and fecal blood (scored as 0–12). **(C)** Body weight curve of different groups. **(D)** Representative gross photographs of mouse colon, **(E)** the colon length and **(F)** the spleen weight in different groups were measured. **(G)** Serial sections from colon tissue stained with H&E. **(H)** Histopathological (HAI) scores of colons from mice with DSS-induced experimental chronic colitis. The pathology images were selected to show the most typical one for each group. DSS, dextran sulfate sodium; H&E, hematoxylin and eosin. ns, p > 0.05.

To investigate the role of BAFF in the experimental colitis model, we observed the effects of BAFF blockade on the inflammation of DSS-induced colitis mice. Modeling and interventions are shown in **Figure 2A**. The DAI scores and body weight loss (**Figures 2B, C**) improvement were observed in anti-BAFF antibody group compared with those treated with DSS alone, reaching statistical significance from day 11. In addition, as vital markers of colitis, colon shortening (**Figures 2D, E**) was greatly protected and spleen weight (**Figure 2F**) was reduced after intraperitoneal injection of BAFF monoclonal antibody in DSS mice, compared to the DSS group. Anti-BAFF group had a lighter spleen weight than DSS group. H&E staining indicated the colonic structure was more severely damaged in the DSS group with immunoglobulin G1 (IgG1) isotype control antibody-treated, characterized by infiltration of inflammatory cells in the lamina propria and loss of crypts, whereas BAFF blockade administration significantly ameliorated structural damage and colonic inflammation (**Figure 2G**). These findings suggest that BAFF blockade can effectively alleviate intestinal inflammation in DSS-induced colitis mice. These results were further confirmed by histological scoring (**Figure 2H**).

### BAFF Blockade Downregulates Inflammatory Cytokine Levels In DSS-Induced Colitis Mice

**Figure 3:**
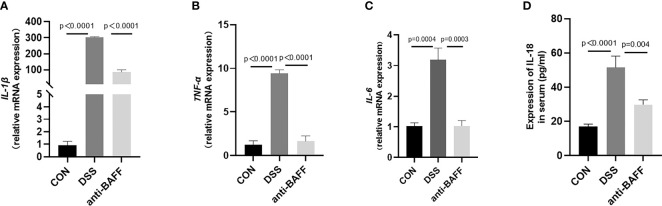
Anti-BAFF antibody decreased colon levels of pro-inflammatory cytokines following DSS exposure. **(A–D)** Total mRNA was extracted from colonic samples to determine the mRNA expression of IL-1β, TNF-α, and IL-6 by RT-qPCR, and IL-18 of serum concentrations by ELISA.

Excess inflammatory response is associated with systemic inflammatory injury and poor outcome in IBD, namely, IL-1β, TNF-α, IL-6, and IL-18. The expressions of cytokines in colon tissues or serum from all groups were measured with RT-qPCR or by ELISA. As exhibited in **Figure 3**, BAFF blockade treatment resulted in a significant reduction in the colon concentrations of IL-1β, TNF-α and IL-6 or serum concentrations of IL-18 after DSS treatment. These results indicate that BAFF antibody treatment improves the systemic inflammatory response in DSS-induced colitis.

### BAFF Blockade Suppresses NLRP3 Inflammasome Expression *Via* NF-κB Signaling *In Vivo*

**Figure 4:**
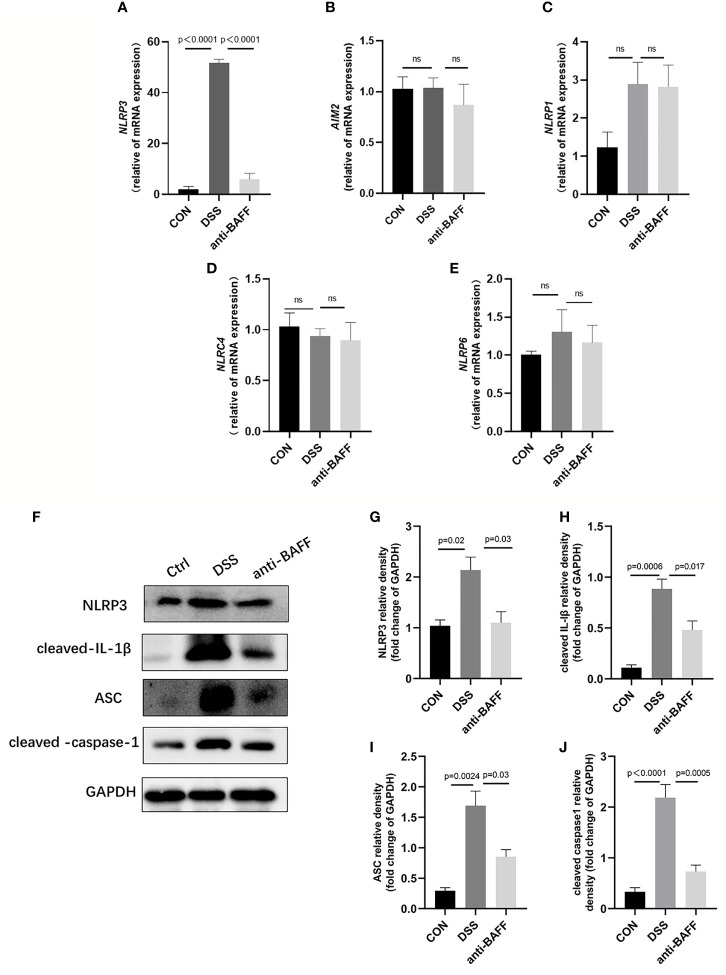
Anti-BAFF antibody decreased colon level of NLRP3 inflammasome in DSS-induced experimental chronic colitis in mice. **(A–E)** Total mRNA was extracted from colonic samples to determine the mRNA expression of NLRP3, AIM2, NLRP1, NLRC4 and NLRP6 by RT-qPCR. **(F)** Representative immunoblot bands for the NLRP3, ASC, cleaved-IL-1β and cleaved-caspase-1 proteins. GAPDH was used as a loading control. **(G–J)** Histogram of relative expression of NLRP3, ASC, cleaved-IL-1β and cleaved-caspase-1 (n = 6–9 per group). NLRP3, NOD-like receptor protein 3, AIM2, absent in melanoma 2, NLRP1, NLR family, pyrin domain containing 1, NLRC4/IPAF, IL-1β converting enzyme protease activating factor, NLRP6, NOD-like receptor family pyrin domain containing 6. ns, P > 0.05. Ctrl, CON.

**Figure 5:**
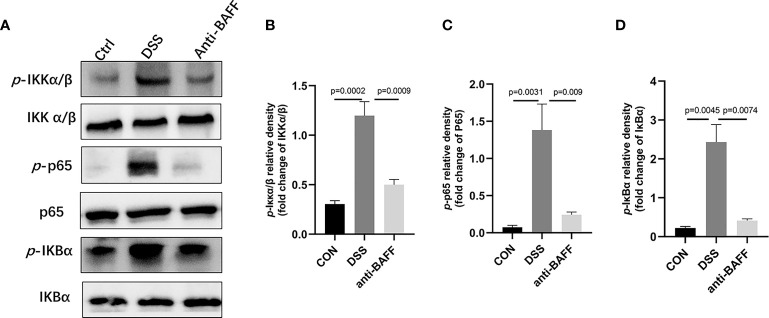
Anti-BAFF antibody inhibited the activation of NF-κB in DSS-induced experimental chronic colitis in mice. **(A)** Representative WB analysis of phospho-IKKα/β, IKKα/β, phospho-NF-κB p65, NF-κB p65, phospho-IKBα, IKBα in colon tissues. **(B)** Histogram of relative expression of p-IKKα/β in colon tissues (n = 6–8 per group). **(C)** Histogram of relative expression of p-p65 in colon tissues (n = 6–9 per group). **(D)** Histogram of relative expression of p-IKBα in colon tissues (n = 6–9 per group). Ctrl, CON.

The NLRP3 inflammasome is an intracellular complex triggering inflammatory responses during IBD, which predominantly activated in macrophages (24). We first examined the gene expression of several common inflammasome (NLRP3, AIM2, NLRP1, NLRP6, NLRC4) by RT-qPCR.We found that only NLRP3 gene expression was significantly increased in the DSS group, and BAFF antibody injection significantly inhibited NLRP3 gene expression. (**Figures 4A–E**). Therefore, we examined the effects of BAFF blockade on NLRP3 inflammasome. The protein expression of NLRP3 increased, followed by increasing level of ASC, cleaved IL-1β, cleaved caspase-1 in the DSS group, compared with the control group (**Figures 4F–J**). BAFF antibody treatment significantly suppressed the protein level of NLRP3, ASC, cleaved IL-1β and cleaved caspase-1. These results suggest that BAFF blockade improves DSS-induced colitis by suppressing the NLRP3 inflammasome. To conform whether the NF-κB signaling pathway is involved in BAFF-mediated regulation of NLRP3 inflammasome, NF-κB signaling activation status was tested by western blot. The levels of *p*-IKKα/β/IKKα/β, *p*-p65/p65 and *p*-IKBα/IKBα were significantly elevated in the DSS group. Nevertheless, anti-BAFF intervention remarkably reduced *p*-IKKα/β/IKKα/β, *p*-p65/p65 and *p*-IKBα/IKBα ratios (**Figure 5**). In all words, these results reveal that anti-BAFF blockade inhibits NLRP3 expression partly *via* reducing NF-κB signaling pathway. NLRP3 serves as one of the core components of the NLRP3 inflammasome, and we hypothesize that BAFF affects the NLRP3 inflammasome in mice with colitis.

### BAFF Blockade Inhibits the Release of Inflammatory Cytokines *In Vitro*

**Figure 6:**
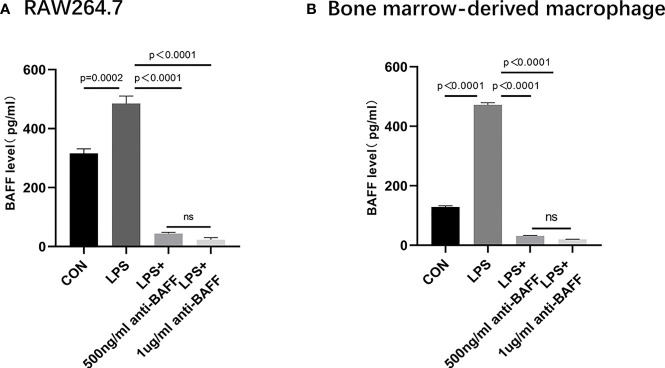
Secretion of BAFF in RAW 264.7 cells and bone marrow derived macrophages (BMDMs). RAW264.7 cells and BMDMs were primed with LPS (1 μg/ml) for 6 h. Anti-BAFF antibody or mouse immunoglobulin G1 (IgG1) isotype control antibody (500 ng/ml or 1 ug/ml) was added to RAW 264.7 and BMDMs at the same time with LPS. **(A)** Detection of BAFF by ELISA in RAW264.7 cells. **(B)** Detection of BAFF by ELISA in BMDMs. ns, P > 0.05.

**Figure 7:**
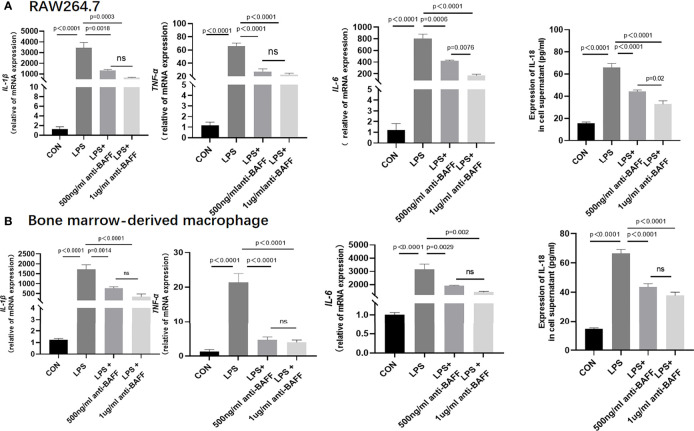
Anti-BAFF antibody plays an anti-inflammatory role in RAW 264.7 cell and BMDM. **(A)** RAW 264.7 cells were intervened with mouse anti-BAFF antibody or mouse immunoglobulin G1 (IgG1) isotype control antibody at 500 ng/ml or 1 ug/ml in the presence of LPS 1 ug/ml for 6 **(h)** The mRNA level of IL-1β, TNF-α, and IL-6 was measured by RT-qPCR, IL-18 was measured in cell supernatant by ELISA. **(B)** Bone Marrow Derived Macrophages were obtained and were treated with mouse anti-BAFF antibody at 500 ng/ml or 1 ug/ml in the presence of LPS 1 ug/ml for 6 h, pulsed with ATP (2 mM, Sigma Aldrich, USA) for 30 min. The mRNA level of IL-1β, TNF-α and IL-6 was measured by RT-qPCR, IL-18 was measured in cell supernatant by ELISA. ns, P > 0.05.

In order to have a more comprehensive understanding of the anti-inflammatory effect of BAFF blockade in macrophages, *in vitro* experiments were performed using RAW264.7 cells and BMDMs. Firstly, we detected a remarkable increase about BAFF levels in the supernatant of LPS-induced RAW264.7 cells and BMDM cells compared to the control, BAFF-neutralizing antibody treatment significantly inhibited LPS-induced BAFF expression (**Figure 6**). Then we elucidated whether BAFF blockade also affected the production of cytokines in RAW264.7 cells and BMDMs. After stimulation with lipopolysaccharide (LPS), anti-BAFF blockade treatment remarkably decreased the expression of IL-1β, TNF-α, IL-6, and IL-18 in RAW264.7 cells and BMDMs by RT-PCR or ELISA (**Figure 7**). Taking all these together, BAFF blockade attenuates the inflammatory status in LPS-pretreated RAW264.7 cells and BMDMs, which are consistent with the experiment *in vivo*.

### BAFF Blockade Inhibits NLRP3 Inflammasome *Via* NF-κB Signaling Pathway *In Vitro*

**Figure 8:**
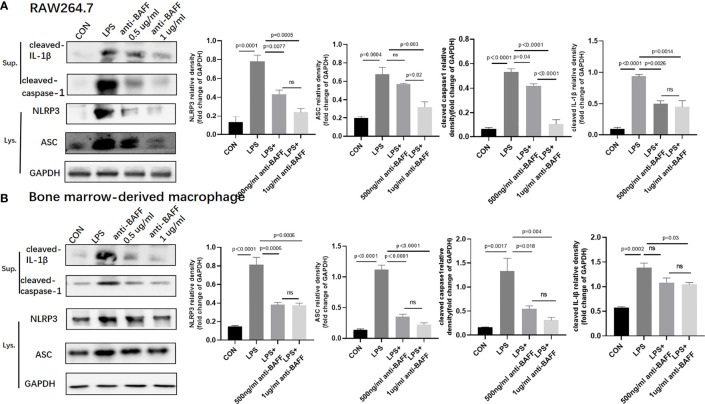
Anti-BAFF antibody inhibited NLRP3 inflammasome protein in RAW264.7 cells and bone derived macrophages (BMDMs). RAW264.7 cells and BMDMs were primed with LPS (1 ug/ml) for 6 h. Anti-BAFF antibody or mouse immunoglobulin G1 (IgG1) isotype control antibody at 500 ng/ml or 1 ug/ml in the presence of LPS 1 ug/ml for 6 h, then BMDMs pulsed with ATP (2 mM, Sigma-Aldrich, MO, USO) for 30 min. **(A, B)** Representative immunoblot bands for the NLRP3, ASC, cleaved-IL-1β, cleaved-caspase-1 proteins. GAPDH was used as a loading control. ns, P > 0.05.

**Figure 9:**
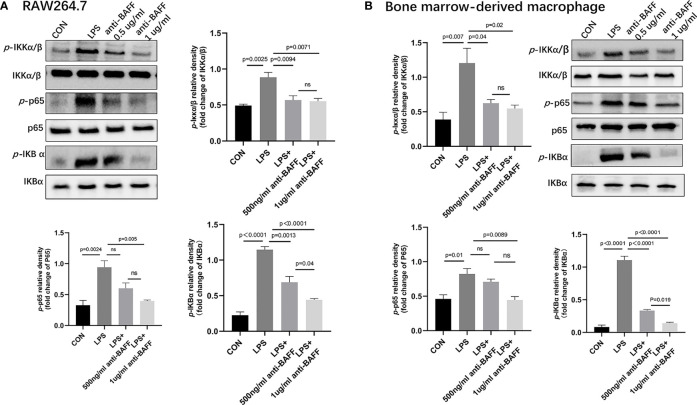
Anti-BAFF antibody inhibited NF-κB signaling pathways in RAW264.7 cells and bone derived macrophages (BMDMs). RAW264.7 cells and BMDMs were primed with LPS (1 ug/ml) for 6 h, then BMDMs pulsed with ATP (2 mM, Sigma-Aldrich, MO, USA) for 30 min. Anti-BAFF antibody or mouse immunoglobulin G1 (IgG1) isotype control antibody at 500 ng/ml or 1 ug/ml in the presence of LPS 1 ug/ml for 6 h. **(A, B)** Representative immunoblot bands for the *p*-IKKα/β, IKKα/β, *p*-p65, p65, *p*-IKBα, IKBα proteins. ns, P > 0.05.

Since IL-1β, TNF-α, IL-6, and NLRP3 were upregulated in the inflamed colonic tissues and profoundly decreased after BAFF blockade, we assessed the effect of BAFF blockade on the activity of NF-κB signaling pathway and NLRP3 inflammasome in RAW264.7 cells and BMDMs. BAFF blockade significantly reduced NLRP3, ASC, cleaved IL-1β and cleaved caspase-1 by western blot in LPS-induced RAW264.7 cells and BMDMs (**Figure 8**). Next we tested whether the NF-κB signaling pathway is involved in BAFF-mediated regulation of NLRP3 inflammasome in RAW264.7 cells and BMDMs. The levels of *p*-IKKα/β, *p*-p65, and *p*-IKBα of the above cells were significantly elevated by western blot, while treatment of BAFF blockade noticeably reduces NF-κB related proteins coherent with findings *in vivo* (**Figure 9**). In conclusion, BAFF blockade intervention suppresses NLRP3 expression through NF-κB signaling pathway inhibition *in vivo* and *in vitro*.

### LPS Synergizes With BAFF to Promote Inflammatory Cytokines Release in RAW264.7 Cells

**Figure 10:**
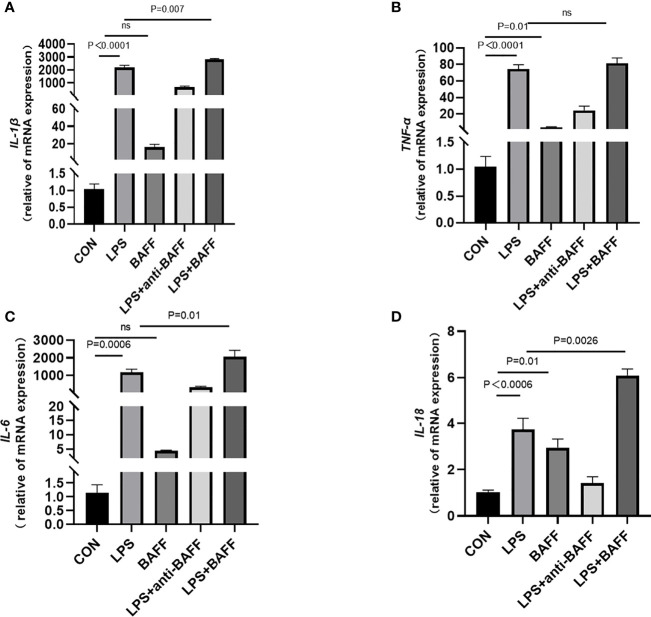
LPS synergizes with BAFF to promote RAW264.7 cells inflammatory cytokines secretion. RAW264.7 cells were primed with LPS (1 ug/ml) for 6 h. Anti-BAFF antibody or mouse immunoglobulin G1 (IgG1) isotype control antibody at 1 ug/ml, recombinant mouse BAFF at 250 ng/ml in the presence of LPS 1 ug/ml for 6 h. **(A–D)** The mRNA level of IL-1β, TNF-α, IL-6, and IL-18 was measured by RT-qPCR. ns, P > 0.05.

**Figure 11:**
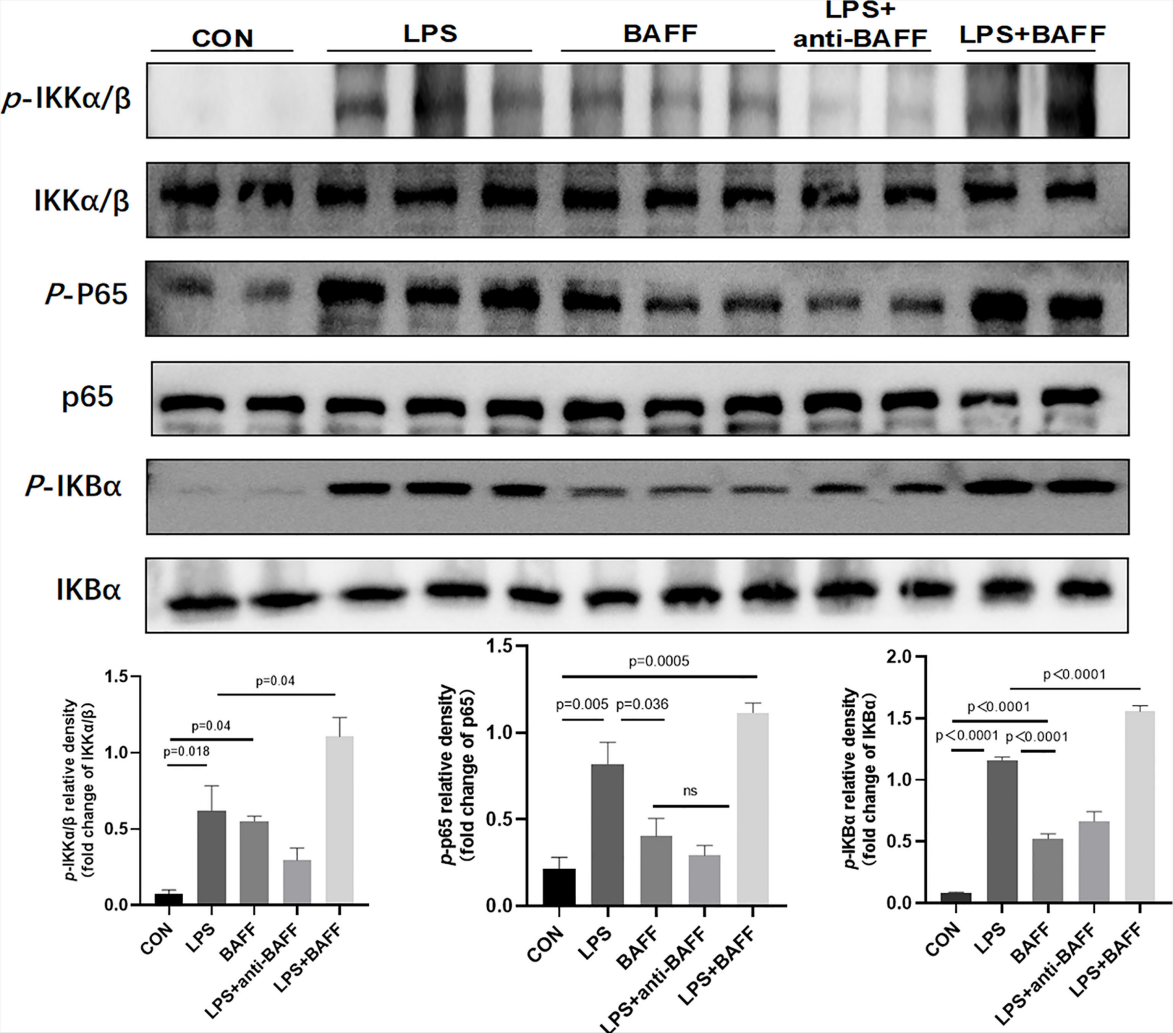
LPS synergizes with BAFF to promote RAW264.7 cells the expression of NF-κB signal pathway. RAW264.7 cells were primed with LPS (1 ug/ml) for 6 h. Anti-BAFF antibody or mouse immunoglobulin G1 (IgG1) isotype control antibody at 1 ug/ml, recombinant mouse BAFF at 250 ng/ml in the presence of LPS 1 ug/ml for 6 h. Representative immunoblot bands for the *p*-IKKα/β, IKKα/β, *p*-p65, p65, *p*-IKBα, IKBα proteins.

Since inflammatory cytokines, NLRP3 inflammasome, and NF-κB signaling pathway were upregulated after anti-BAFF antibody treatment *in vivo* and *in vitro*, we assessed the effect of recombinant mouse BAFF on inflammatory agents and NF-κB signaling pathway. Recombinant mouse BAFF alone neither significantly increased IL-1β, TNF-α, and IL-6 in gene level nor increased NF-κB signaling pathway in RAW264.7 cells. Interestingly, recombinant mouse BAFF together with LPS remarkably increased the above mentioned inflammatory cytokines and key proteins of the NF-κB pathway (*p*-IKKα/β/IKKα/β, *p*-P65/P65, *p*-IKB-α/IKB-α) (**Figures 10**, **11**).

## Discussion

To our knowledge, this is the first report to study the mechanism underlying BAFF in IBD models *in vivo* and *in vitro*. We elucidate that BAFF is probably involved in IBD and made the following conclusions: (1) BAFF is overexpressed in colitis mice, LPS-induced RAW264.7 cells and BMDMs. (2) BAFF blockade improves inflammatory status, body weight loss, spleen weight, DAI score, colonic length and colonic pathological damage in colitis mice and improves inflammation in LPS-induced RAW264.7 cells and BMDMs. (3) BAFF blockade improves colitis by inhibiting NF-κB signaling pathway and NLRP3 inflammasome activation.

Accumulating evidence shows that BAFF is a crucial regulatory mediator in several inflammatory diseases. Studies show that BAFF is significantly elevated in the blood circulation in SLE patients, with higher levels in active than inactive patients, making a strong correlation with high levels in BAFF and poor prognosis among SLE patients (25). Research related to autoimmune hepatitis (AIH) has shown that serum BAFF levels are significantly higher in patients with AIH, compared to hepatitis patients and healthy controls, with positive correlations to AST, ALT, TBIL, and soluble CD30, implying BAFF contributes to liver injury in patients with AIH (26). BAFF expression was significantly higher in small pulmonary artery endothelial cells, alveolar epithelial cells, and alveolar macrophages in COPD patients, while circulating BAFF concentrations were higher in patients who smoked compared to non-smokers, suggesting that BAFF was associated with impaired lung function, hypoxia severity in COPD patients (27–29). We previously found elevated BAFF in serum, stool and colon tissue of adults with ulcerative colitis and Crohn’s disease, and serum BAFF levels correlated with disease activity, ESR, TNF-α, and IL-1β in ulcerative colitis patients (8). Another study discovered that BAFF levels in serum and feces were higher in pediatric ulcerative colitis and Crohn’s disease than in stress ulcers and healthy controls, correlating significantly with fecal calprotectin (9). This study detected significantly elevated levels of BAFF in serum and colonic tissues from DSS-induced chronic colitis mouse model and in cell supernatants of the LPS-induced RAW264.7 cells and BMDM, consistent with the findings of previous studies in human tissue, serum and fecal samples.

Elevated BAFF levels in colitis models indicate that BAFF may be involved in the pathogenesis with colitis. Several inflammatory models suggest that BAFF exhibits pro-inflammatory effects. BAFF significantly promotes the secretion of serum IL-1β, TNF-α, IL-6 and other inflammatory factors in the LPS-induced sepsis model whereas neutralization of BAFF markedly inhibits pro-inflammatory factor release (30). In rheumatoid arthritis (RA) model, BAFF promoted B-cell survival and also secretion of pro-inflammatory factors IL-6 and IL-8 in mice, consistent with the result in synovial fibroblasts co-cultured with INF-γ (31). Another rheumatoid arthritis model in mice using BAFFR siRNA-coated nanoparticles targeted to deliver B cells after intravenous injection reduced arthritis scores, serum anti-collagen IgG levels, and improved rheumatoid arthritis symptoms in collagen induced arthritis (CIA) mouse models (32). The anti-BAFF monoclonal antibody effectively reduced BAFF levels *in vivo* and *in vitro* and improved body weight loss, DAI score, colon length, spleen weight and colonic pathological damage in DSS-induced colitis mice, suggesting that BAFF blockade is effective against IBD.

BAFF promotes elevated inflammatory factors such as IL-1β, IL-6, IL-23, and TGF-β in *Helicobacter pylori*-associated chronic gastritis (33–35). TNF-α, IL-1β, and IL-6 levels are significantly elevated and are key intestinal proinflammatory cytokines that play an important role in both the pathogenesis and progression of IBD (36). Our study showed that blockage of BAFF activity effectively inhibited IL-1β, TNF-α, and IL-6 gene expression in colonic tissues from DSS-induced colitis mice. We also observed that BAFF neutralizing antibody treatment efficiently inhibited IL-1β, TNF-α, IL-6 gene expression in LPS-induced RAW264.7 cells and BMDMs. These results suggest that BAFF is an important regulator in the immune system.

Several studies demonstrated that inflammasome are mainly activated by macrophages following internal and external stimulation (e.g., PAMP or DAMP). The NLRP3 inflammasome consists of NLRP3, ASC, and caspase-1 and plays a central role in PAMP/DAMP-induced intrinsic immunity. NLPR3 activates caspase-1 after triggering by PAMP/DAMP, and activates NF-κB signaling to induce IL-1β and IL-18 transcription and maturation. Reports have shown that BAFF binding to BAFFR further induces NLRP3 inflammasome initiation and activation signaling *via* activation of the NF-κB pathway to maintain primary B cell and B lymphoma cell line survival and homeostasis *in vivo*. In this article we explored whether BAFF exerts a regulatory role on inflammasome (20, 37). In this study, we detected several common NOD-like receptor and found only NLRP3 gene expression was significantly elevated in DSS-induced colitis model, while NLRP3 gene expression and NLRP3 inflammasome key protein (NLRP3, ASC, cleaved-IL-1β, cleaved-caspase1) were significantly inhibited in colonic tissue of colitis mice and LPS-induced RAW264.7 cells and BMDMs after BAFF blockade.

To activate NLRP3 inflammasome, the first signal is the PAMP-mediated to activate the NF-κB signaling pathway, which promotes the accumulation of NLRP3, pro-IL-1β and pro-IL-18; the second signal is the DAMP-mediated recruitment *via* ASC proteins to form caspase-1 after catalytic processing (38). NF-κB signaling pathway plays an important regulatory role in regulating NLRP3 inflammasome (39). Our study confirmed that BAFF blockade significantly inhibited NF-κB pathway proteins (*p*-IKKα/β/IKKα/β, *p*-p65/p65, *p*-IKBα/IKBα)in DSS-induced chronic colitis mouse model and LPS-induced RAW264.7 cells and BMDMs. BAFF blockade treatment has protective effects in colitis, probably related to the inhibition NF-κB signaling pathways and also the NLRP3 inflammasome. However, there are still several limitations in this study. Firstly, this study only examined BAFF in DSS-induced colitis model and LPS-induced RAW264.7 cells and BMDMs. BAFF in other models of inflammatory bowel disease such as TNBS colitis model and IL-10 knockdown colitis model and also human THP-1 cells remains to be explored further. Furthermore, we focused only on macrophage-inflammasome related functions in this study, and BAFF in other mechanisms of colitis is not elucidated.

### Conclusions

In this study, we unveiled that BAFF is increased in the circulation system and colon tissue in DSS-induced colitis mice. Besides, our findings showed that macrophages are one of the important sources of BAFF. Neutralizing BAFF can ameliorate colitis by reducing inflammation, inhibiting NF-κB and NLRP3 signaling pathways. Hence our findings provide a promising therapeutic targ\et and focus for clinical inflammatory bowel disease.

## Data Availability Statement

The raw data supporting the conclusions of this article will be made available by the authors, without undue reservation.

## Ethics Statement

The animal study was reviewed and approved by the Experimental Animal Ethics Committee, Tongji Medical College, Huazhong University of Science and Technology.

## Author Contributions

Designed the study: YZ and YF. Performed the experiments: YZ, MHT, CYC, and QYF. Analyzed the data: YZ, MHT, and XZ. Drafted the manuscript: YZ. Revised the manuscript: YF and GC. All authors listed have made a substantial, direct, and intellectual contribution to the work and approved it for publication.

## Funding

This study is funded by the National Natural Science Foundation of China [grant numbers 82070572, 81570501, and 81770554].

## Conflict of Interest

The authors declare that the research was conducted in the absence of any commercial or financial relationships that could be construed as a potential conflict of interest.

## Publisher’s Note

All claims expressed in this article are solely those of the authors and do not necessarily represent those of their affiliated organizations, or those of the publisher, the editors and the reviewers. Any product that may be evaluated in this article, or claim that may be made by its manufacturer, is not guaranteed or endorsed by the publisher.
